# Community-dwelling older adults’ satisfaction and experiences with two non-surgical caries treatments: a mixed methods study

**DOI:** 10.3389/froh.2026.1814583

**Published:** 2026-05-11

**Authors:** Gloria C. Bales, Shelley Curtan, Kelly Hickey, Annabelle Cohen, Ronia Hightower, Farida Ejaz, Suchitra Nelson

**Affiliations:** 1 Department of Community Dentistry, Case Western Reserve University School of Dental Medicine, Cleveland, OH, United States; 2 Consultant, Aging Research, Cleveland, OH, United States

**Keywords:** caries, dental anxiety, older adults, satisfaction, silver diamine fluoride

## Abstract

**Introduction:**

Silver Diamine Fluoride (SDF) and Atraumatic Restorative Treatment (ART) are effective non-surgical treatments for caries, but few studies have assessed the satisfaction of these treatments with older adults. Therefore, the objective of this study was to evaluate older adults’ satisfaction with SDF and ART.

**Methods:**

The participants were part of a larger cluster-randomized clinical trial to investigate comparative effectiveness of SDF vs. ART. They were ≥62 years of age and lived independently in subsidized housing facilities. A mixed methods approach was used to evaluate satisfaction: 1) quantitatively utilizing a 6-item satisfaction survey completed by all participants at the final follow-up visit at 52 weeks; and 2) qualitatively by focus group discussions with randomly selected participants. Multiple data sources were used to increase data triangulation. Bivariate analyses were completed for surveys, and a thematic analysis was conducted for focus groups. The discussions were transcribed, coded, and analyzed for emerging themes.

**Results:**

Overall, 485 participants completed the satisfaction survey, and three focus groups were conducted with a total of 20 participants (*n* = 6–7 per group). The survey results indicated 97% overall satisfaction with SDF and ART. Over 90% of the participants were also satisfied with the treatments’ ability to treat or prevent caries, ease, lack of side effects, and they were more likely to choose non-surgical treatments over those requiring drilling or needles. Focus groups further supported these findings, and five themes were identified: 1) determinants of participant satisfaction, 2) factors of dissemination, 3) benefits of non-invasive caries treatments, 4) issues related to delayed dental care, and 5) facilitators for receiving dental care. Focus group participants reported treatments as painless, convenient, relieved dental anxiety, effective, and would recommend to other older adults.

**Conclusion:**

These findings indicate high satisfaction levels for SDF and ART treatments in low-income community-dwelling older adults, and that they can be delivered in clinical and community settings.

## 1 Introduction

Treatment satisfaction can be based on many factors such as efficacy, side effects, impact on daily life, and route of delivery (1). Using a validated instrument such as the Treatment Satisfaction Questionnaire for Medication can provide insight on satisfaction or when a change in treatment may be needed (1). This instrument has been validated using a variety of conditions such as diabetes, depression, infectious disease, and arthritis (1) and has been used to evaluate treatment satisfaction in those with rheumatoid arthritis and in hypertensive patients (2, 3).

Adults in the U.S. aged 65 and older with lower incomes have higher rates of untreated decay and tooth loss (4) compared to those with incomes above the federal poverty level. Older adults face barriers to dental care including lack of insurance coverage (5), changes in dentition and increased sensitivity to local anesthetics as used in conventional restorations (6), lack of transportation (7), negative dental experiences (7), and dental anxiety (8). Different treatment options are available for tooth decay, i.e., Silver diamine fluoride (SDF) and Atraumatic Restorative Treatment (ART) are both effective non-surgical approaches (no drilling or local anesthesia) to caries arrest and prevention (9, 10) compared to conventional restorations using a surgical approach. ART is a simple dental strategy applied by dental professionals by gently removing decayed debris and restoring the tooth with glass ionomer cement, while SDF is a topical application brushed on the teeth (11) and may also be applied by non-dental professionals (12). In a recent clinical trial, biannual applications of SDF were found to be noninferior for caries arrest compared to ART in older adults (13), providing support for broader SDF implementation. Benefits of SDF include its low cost and easy topical application (11), and one study found SDF treatments to be a feasible solution for those with mobility limitations (14). ART can be applied without the use of a drill to remove decay, and it has been shown to have a lower cost than conventional treatments (15). ART has been utilized for underserved communities with low resources due the reduced cost of equipment and materials (16). Additionally, ART has been found to be a preferable approach for those with disabilities (17).

There are few prior studies reporting on the satisfaction of SDF and ART in older adults (14, 18). The existing satisfaction studies are based on single SDF applications, unlike this study which utilizes biannual applications of SDF as previously suggested for caries arrest (13, 19). In one adult study (ages: 25–60 years), the mean satisfaction score for a single application of SDF was higher at 4.7 ± 0.5, while fluoride varnish was 3.5 ± 0.8 (18). One qualitative study, with older adults receiving SDF for root caries, reported that participants viewed treatment favorably if suggested by a trusted dentist, the tooth stain due to SDF was not visible such as with anterior teeth, and it was important for tooth preservation (14). Few barriers have been reported for SDF treatments but may include: negative perceptions of the staining side effect and a general lack of knowledge about root caries and SDF as a treatment (14).

Parent satisfaction for ART has been reported in children and young adults with disabilities (17) but is lacking among older adults. These studies compare ART to conventional restoration for caries treatment (17, 20). In one study, parents or caregivers reported higher satisfaction with ART compared to conventional restorations in children and young adults with disabilities one year after treatment (17), while in a qualitative study, parents reported that with ART, they were able to establish a relationship with the care team (20). The authors suggest that ART is an alternative treatment option compared to conventional restorations (20), and patient barriers were not reported in these studies (17, 20).

There is a paucity of literature on satisfaction for SDF and ART caries treatments in older adults. This mixed methods study aims to evaluate community-dwelling older adults' satisfaction with two non-surgical caries treatments: 1) quantitatively with satisfaction surveys from older adults who participated in a cluster randomized clinical trial (cRCT) treated with SDF or ART; and 2) qualitatively in focus groups of randomly selected participants in the cRCT exploring satisfaction, experiences, recommendations, barriers or challenges, and resources regarding their treatments.

## 2 Methods

### 2.1 Study design

This study utilized satisfaction surveys and focus group data from a larger cluster-randomized clinical trial (cRCT) that compared the non-inferiority of SDF vs. ART (21). The trial was conducted in 33 subsidized housing facilities. An explanatory sequential mixed methods approach was used as the data from the satisfaction survey were collected during the last follow-up visit (22). After final visits were complete, randomly selected older adults could participate in a focus group. Multiple data sources were used to obtain triangulation (23). The study was approved by the Institutional Review Board of Case Western Reserve University and registered in clinicaltrials.gov (NCT03916926). Written consent was obtained prior to study participation. This study conforms to GRAMMS (Good Reporting of Mixed Methods Study) guidelines.

### 2.2 Setting and subjects

Recruitment for the cRCT took place from September 2019 to April 2023. Participants were from publicly subsidized housing sites [Housing and Urban Development (HUD) Section 202; low-income housing voucher programs] located in Cuyahoga, Lorain, and Summit Counties in NE Ohio (*n* = 33) and lived independently. Inclusion criteria for the cRCT included: age ≥62, lived at a participating housing site, willingness to comply with study procedures, and had at least one root or coronal caries lesion with an International Caries Detection and Assessment System (ICDAS) (24) active lesion score ≥3. Housing sites were randomized to the SDF (intervention) or ART (control) arms (13) and participants within a site received the same treatment.

#### 2.2.1 Intervention

Dental exams and treatments in the cRCT were performed by trained and calibrated dental hygienists under a supervising dentist at each housing site in a designated area. Exams were conducted at baseline, 26 weeks, and 52 weeks. Portable equipment was used at the housing facility for the exams and treatments. In the intervention arm, participants with an active ICDAS lesion score of ≥3 received bi-annual topical 38% SDF at baseline and at 26 weeks (13). For the control arm, participants with the same active lesion score received ART in conjunction with bi-annual 2.5% topical fluoride varnish at baseline and 26 weeks. The lesion was cleaned to remove any debris, and then restored with a resin-reinforced glass ionomer cement (13).

For this study, participants who completed the 52-week dental exam, treatment, and satisfaction survey were included in the quantitative analysis. For the qualitative objective, randomly selected participants from each of the treatment groups were asked to participate in the focus groups after the final 52-week visit.

### 2.3 Socio-demographics

All participants completed the sociodemographic questions at baseline. The questions were adapted from the Third National Health and Nutrition Examination Survey (NHANES III) (25) including: sex (male, female); race (American Indian or Alaskan, Asian, Black or African American, Hawaiian or Pacific Islander, More than one race, and White); mean age; and education [Grade School or Less (0–8 Years of School), Some High School or Completed High School (9–12 Years), Some College, Completed College, and Masters' Degree or Higher].

### 2.4 Satisfaction survey

All participants completed the self-reported modified Treatment Satisfaction Questionnaire for Medication (TSQM) (1) and Satisfaction with New Treatment for cavities (26) at the final 52-week visit. The TSQM has been previously validated (1) and four questions were modified for dental (treatments' ability to treat or prevent caries, ease, side effects, and overall satisfaction). Two questions were used for the Satisfaction with New Treatment for cavities from a previous study (preferable to traditional treatments using a local anesthetic or drills) (26). TSQM responses were on a 5-point Likert scale from extremely dissatisfied to extremely satisfied (extremely satisfied and satisfied were collapsed as were extremely dissatisfied and dissatisfied). Responses for the New Treatment questionnaire were categorized on a 4-point Likert scale from extremely unlikely to very likely (very likely and somewhat likely were collapsed as were extremely unlikely and somewhat unlikely).

### 2.5 Focus groups

In the cRCT, housing sites were rolled out at different times during enrollment, and participants completed their final 52-week exam during different years (2 through 5) of the study. For the focus groups, sites and participants (from each site) were chosen randomly as follows: 1) participants must have given consent to be contacted for the focus groups and audio recorded; 2) participants completed their 52-week dental exam and treatment; 3) each year a randomized list was generated by the biostatistician from a database of those meeting the previous criteria 1 and 2; 4) research staff then called participants from this list, and those that were available and willing to participate were scheduled for the focus group. These focus group discussions were conducted in years 2, 4, and 5 of the study and each focus group consisted of different participants. Due to COVID restrictions, the focus group planned to take place in year three was delayed one year. Focus groups were conducted over Zoom due to COVID 19 and logistical restrictions (participants from multiple housing sites). Research staff provided tablets for those not able to use their own device and provided technical support as needed. Participants had no prior contact with the moderator during the cRCT.

Three research team members with content expertise in behavioral science, geriatrics, and epidemiology developed five focus groups questions to better understand lived experiences through open-ended questions with probes using a combination of a phenomenological approach and grounded theory (27–29). These team members had previous experience in qualitative methodology and focus group guide development (29). Key topics included experiences, satisfaction, dissemination, barriers to study treatments, barriers to future dental care, and resources/challenges for older adults receiving dental care. The focus group guide is included in the Supplementary Material.

### 2.6 Data analysis

Descriptive statistics (frequencies, mean, and standard deviation) were used for analysis of satisfaction survey and focus group participant data. Bivariate analyses (*t*-test, *X*^2^) were completed for socio-demographics and the satisfaction survey between the two arms. The analysis was conducted using tidyverse packages in R, version 4.4.2 (R Group for Statistical Computing), RStudio (Posit Software), and version 2024.12.1 (Build 563).

The Theory of Planned Behavior (TPB) and the Consolidated Framework for Implementation Research (CFIR) were used to guide interpretation and alignment of emergent themes from focus group discussions during the analysis stage from participants' experiences and perceptions (30–32). TPB has been used previously to understand intent or willingness to receive dental care and changes in oral health behaviors (33, 34), and this theory was used to guide interpretation of attitudes and willingness towards SDF and ART treatments. The CFIR framework (e.g., relative advantage, outer setting) was used in a previous study to understand determinants (advantages, patient needs) of SDF treatment (35). In this study, the CFIR domains used for interpretation were: innovation (relative advantage), implementation process, inner setting, and outer setting (31) to assess advantages, dissemination, and barriers of SDF and ART treatments, as well as barriers and facilitators for future dental treatment.

For the focus groups, only Zoom audio recordings were used. They were de-identified, transcribed, and verified by a second research team member. Transcripts were kept on a password protected computer. A thematic analysis, using a similar approach as outlined by Braun and Clark (36), was conducted with four coders from the research team. Data was coded initially using the five questions with probes, followed by open coding. Coders then individually categorized the data into patterns and trends. Emerging themes were then discussed collaboratively by the coders and one other research team member (non-coder). The iterative process of engaging with the data guided the development of the themes, and coders agreed that data saturation was achieved when there was an absence of new information. Triangulation among coders enhanced trustworthiness by integrating multiple perspectives in the process (23). Focus group data was coded and analyzed using NVivo 14 (QSR International).

## 3 Results

### 3.1 Satisfaction survey

From the 568 participants enrolled in the cRCT, 485 completed the satisfaction survey with a mean age of 70.07 (6.79). Participants were predominantly female (51%), Black or African American (65.5%), and had completed some or all of high school (56.8%). Significant differences were found in age, sex, and race between the treatment groups. A greater number of females were in the SDF group, while more males were in the ART group. A higher percentage of Black person(s)/people were in both treatment arms compared to other races, although a higher percent was in the ART group (Table 1).

**Table 1:** Socio-demographics for the satisfaction survey and focus group.

Socio-demographic variables for satisfaction survey	Overall	ART	SDF	*p*-value
	*n* = 485	*n* = 193	*n* = 292	
Age [Mean (SD)]	70.07 (6.79)	69.29 (6.32)	70.58 (7.05)	0.04
Sex (%)				
Female	249 (51.4)	85 (44.0)	164 (56.4)	0.01
Male	235 (48.6)	108 (56.0)	127 (43.6)	
Race (%)^a^				
American Indian or Alaskan	1 (0.2)	0 (0.0)	1 (0.3)	0.003
Asian	2 (0.4)	2 (1.1)	0 (0.0)	
Black or African American	312 (65.5)	140 (74.5)	172 (59.7)	
More than one race	19 (4.0)	7 (3.7)	12 (4.2)	
White	142 (29.8)	39 (20.7)	103 (35.8)	
Education (%)				
Grade School or Less	7 (1.5)	3 (1.6)	4 (1.4)	0.662
Some High School/Completed High School	274 (56.8)	116 (60.7)	158 (54.3)	
Some College	127 (26.3)	45 (23.6)	82 (28.2)	
Completed College	60 (12.4)	21 (11.0)	39 (13.4)	
Master's Degree Or Higher	14 (2.9)	6 (3.1)	8 (2.7)	
Socio-demographic variables for Focus Group				
	(*n* = 20)	(*n* = 6)	*n* = 14	
Age [Mean (SD)]	72.5 (9.56)	66.65 (6.72)	75.01 (9.68)	0.072
Sex (%)				
Female	14 (70.0)	4 (66.7)	10 (71.4)	1.000
Male	6 (30.0)	2 (33.3)	4 (28.6)	
Race (%)^a^				
American Indian or Alaskan	0 (0.0)	0 (0.0)	0 (0.0)	0.743
Asian	0 (0.0)	0 (0.0)	0 (0.0)	
Black or African American	5 (26.3)	1 (20.0)	4 (28.6)	
More than one race	1 (5.3)	0 (0.0)	1 (7.1)	
White	13 (68.4)	4 (80.0)	9 (64.3)	
Education (%)				
Grade School or Less	1 (5.0)	0 (0.0)	1 (7.1)	0.264
Some High School/Completed High School	10 (50.0)	2 (33.3)	8 (57.1)	
Some College	2 (10.0)	0 (0.0)	2 (14.3)	
Completed College	6 (30.0)	3 (50.0)	3 (21.4)	
Master's Degree or Higher	1 (5.0)	1 (16.7)	0 (0.0)	

SDF, silver diamine fluoride, ART + FV, atraumatic restorative treatment plus fluoride varnish.

a For race: 1 respondent had a missing value, and there were 0 respondents for Hawaiian or Pacific Islander.

No significant differences in satisfaction survey questions were found between treatment groups, indicating similar satisfaction for SDF and ART. Based on the perception of receiving dental care, 93% of the participants were satisfied with both treatments for arrest and prevention of caries. Most participants reported [96% (SDF); 97% (ART)] that side effects [e.g., staining, metallic taste (SDF), filling not exact tooth color (ART)] did not interfere with overall satisfaction. Getting both treatments was reported as easy or very easy by 99% of participants, and overall satisfaction was 97% (SDF) and 98% (ART). When asked if an anesthetic shot was required for fillings (traditional), 89% (SDF) and 95% (ART) said it was somewhat to very likely they would choose these non-surgical treatments instead. The same question was asked for drilling, and 90% (SDF) and 94% (ART) said that they were somewhat likely to very likely to choose these treatments (Table 2).

**Table 2:** Satisfaction survey.

Questions	Overall	ART	SDF	*p*-value^a^
	*n* = 485	*n* = 193	*n* = 292	
How satisfied or dissatisfied are you with the ability of the treatment to prevent or treat your oral health condition?				
Extremely Satisfied/Satisfied	452 (93.2)	180 (93.3)	272 (93.2)	0.331
Dissatisfied/Extremely Dissatisfied	6 (1.2)	3 (1.6)	3 (1.0)	
Neutral	8 (1.6)	5 (2.6)	3 (1.0)	
Don't Know/Missing	19 (3.9)	5 (2.6)	14 (4.8)	
To what degree have side effects affected your overall satisfaction with the treatment?				
Very Great Extent/Great Extent	4 (0.8)	1 (0.5)	3 (1.0)	0.896
Somewhat	6 (1.2)	2 (1.0)	4 (1.4)	
Very Little/Not at All	468 (96.7)	188 (97.4)	280 (96.2)	
Don't Know/Missing	6 (1.2)	2 (1.0)	4 (1.4)	
How easy or difficult was the treatment in its current form?				
Easy/Very Easy	481 (99.2)	191 (99.0)	290 (99.3)	0.457
Neutral	1 (0.2)	0 (0.0)	1 (0.3)	
Very Difficult/Difficult	0 (0.0)	0 (0.0)	0 (0.0)	
Don't Know/Missing	3 (0.6)	2 (1.0)	1 (0.3)	
How satisfied or dissatisfied are you with this treatment?				
Extremely Satisfied/Satisfied	472 (97.3)	190 (98.4)	282 (96.6)	0.37
Neutral	4 (0.8)	0 (0.0)	4 (1.4)	
Dissatisfied/Extremely Dissatisfied	2 (0.4)	1 (0.5)	1 (0.3)	
Don't Know/Missing	7 (1.4)	2 (1.0)	5 (1.7)	
If you required local anesthetic, such as a Novocain shot to, to do fillings, would you choose the new treatment instead of doing fillings?				
Somewhat Likely/Very Likely	439 (91.1)	182 (94.8)	257 (88.6)	0.067
Extremely Unlikely/Somewhat Unlikely	9 (1.9)	2 (1.0)	7 (2.4)	
Don't Know/Missing	34 (7.1)	8 (4.2)	26 (9.0)	
If your teeth needed to be drilled for fillings, would you choose the new treatment instead of fillings?				
Somewhat Likely/Very Likely	444 (91.5)	181 (93.8)	263 (90.1)	0.342
Extremely Unlikely/Somewhat Unlikely	8 (1.6)	2 (1.0)	6 (2.1)	
Don't Know/Missing	33 (6.8)	10 (5.2)	23 (7.9)	

SDF, silver diamine fluoride, ART + FV, atraumatic restorative treatment.

a Significance at *α* < .05.

### 3.2 Focus groups

Focus groups were between 45 and 49 min in length. For the three focus groups, participants (*n* = 20) were from 10 (6-SDF, 4-ART) of the 33 housing sites (Focus Group 1: *n* = 7, Focus Group 2: *n* = 7, Focus Group 3: *n* = 6). Participants were mainly female (70%), white (65%), completed some or all of high school (50%), and had an average age of 72.5 (9.56). No significant differences were found between treatment groups (Table 1).

Five themes were identified from focus groups (Table 3):

**Table 3:** CFIR domains & TPB constructs with Key topics, themes, and quotes.

CFIR domain/TPB construct	Main question with key topic^a^	Themes	Participant quotes
Innovation (Relative Advantage) & Attitudes	Describe your EXPERIENCE and SATISFACTION levels with the dental treatment (SDF or ART + FV) that you received?	*Theme 1: Determinants of Participant Satisfaction* Research Team: Professional, informative about oral hygiene and resources Treatment: Relieved Fear and Anxiety, Convenient, Easy, Painless, Free, Effective, Helped with previous pain	FG2P3:“…being scheduled…and getting taken care of right away, the convenience of being in the building.” FG1P7: “The treatment made my teeth stop hurting…and so I was very satisfied.”
Implementation Process & Intent (Willingness)	Having received our dental treatment, would you RECOMMEND these treatment options to other older adults?	*Theme 2: Factors of Dissemination* Recommended to others: treatment location and painless Would like to know how to “spread the word” (brochure, etc.) Need information to take to the dentist to get the same treatment	FG3P4: “I’ve been telling everybody when I hear that they gotta go to the dentist…I tell them what I went through, they’re like ‘God, why can’t my dentist do this.’”
Inner Setting	What are the BARRIERS and CHALLENGES that you faced in getting our dental treatment?	*Theme 3: Benefits of Non-Invasive Cavity Treatments* Onsite location, no wait time, minimal treatment time, no cost Follow-ups during COVID No drilling, needles, or pain, and less anxiety	FG3P4: “I didn’t have any barriers; you came to my building. It made it very accessible for me.” FG3P5:“No pain. I was very happy with no needles or drills.”
Outer Setting	What would the BARRIERS and CHALLENGES be for you or other older adults in your community to receive dental treatment?	*Theme 4: Issues Related to Delayed Dental Care in Older Adults* Transportation, mobility, cost, insurance, wait time, dental fear/anxiety, traumatic experiences, technology limitations, motivation Treated poorly due to coverage type or age Dentist not relatable and/or trustworthy	FG1P1:“And these younger practitioners and dentists don't like to deal with us.” FG1P5: “But I can't afford to pay for transportation to go to the dentist.”
Inner Setting	What are the RESOURCES available to you to seek dental care in the future?	*Theme 5: Facilitators for Receiving Dental Care in Older Adults* Facility (e.g., social workers, transportation) Family (financial, transportation) Dental insurance and payment plans Information resources (e.g., insurance catalog, internet) Suggested resource-mobile dentist	FG3P6:“Some places want you to pay the big portions first, then put you on a payment plan for the rest.” FG2P4:“…my family and…they were willing to help, financially.”

SDF, silver diamine fluoride; ART + FV, atraumatic restorative treatment plus fluoride varnish; CFIR, consolidated framework for implementation research; TPB, theory of planned behavior; FG, focus group; P, participant.

a Questions are abbreviated in the table.

The CFIR domain, “innovation (relative advantage)”, along with attitudes, from TPB, was used to guide the development of theme 1, *determinants of participant satisfaction*. Participants reported satisfaction within two main categories, the research team and the treatment. They described the team as professional and informative about oral hygiene, and the relative advantages of the treatment as convenient (completed at their housing site), relieved fear and anxiety, and pain free. Participants thought the treatment was effective, and one mentioned it helped with previous pain.

“I’ve been very satisfied. It has been very successful for me….and needle free. I have extreme anxieties when it comes to a needle.”—FG3 (Focus Group 3) P6 (Participant 6)

Theme 2, *factors of dissemination*, was based on the CFIR domain “implementation process” and intent or willingness from TPB. All participants reported that they recommended their treatment to others and expressed willingness to receive the treatment in the future. One participant wanted to “spread the word” to more residents, while one asked for information to take to their own dentist for the same treatment.

“I have recommended to others in the building.”—FG1P7

Theme 3, *benefits of non-invasive cavity treatments*, and theme 5, *facilitators for receiving dental care in older adults,* were developed using the “inner setting” domain of the CFIR framework. For theme 3, benefits of the two treatments were described as: onsite (housing facility), no wait time, minimal time in the dental chair, and free. One participant boasted that follow-ups were completed during COVID, while one experienced less dental anxiety due to no drilling, needles, or pain. All participants reported no barriers in receiving treatments.

“So there were no barriers at all. They said ‘you’d be the first person in’, so I said “that's great”.”—FG2P1

For theme 5, resources or facilitators for future dental care varied by participant with some reporting none. One participant stated that family was a resource (transportation, financial), and a few reported social workers or transportation provided by their facility. Some reported having dental coverage or using payment plans as resources, and a suggested facilitator to future dental care was having a mobile dentist continue to deliver treatment at their housing facility.

“Sometimes I think, if we can have some sort of a mobile dental lab.”—FG1P4

Theme 4, *issues related to delayed dental care in older adults*, was developed based on the “outer ring” domain of CFIR. For future dental care, even though some reported dental coverage, many mentioned cost and lack of coverage as a barrier for care outside of the intervention. Other barriers included: transportation, mobility (walking in/out of dental offices), wait times, technology, dental anxiety, and negative experiences (treated poorly due to coverage and age). One participant thought their dentist was not relatable, while another lacked motivation to go to a dentist outside of the study.

“Well, I don't have Medicaid so I had to pay out of pocket so…I just have to look around and see who I could get and hoping, I can find it cheaper.”—FG1P2

## 4 Discussion

This is the first study, to our knowledge, that assesses satisfaction with SDF and ART for the treatment and prevention of caries in community-dwelling older adults using a mixed methods approach. Both quantitative satisfaction surveys and qualitative focus group discussions were used for exploration of experiences, recommendations, and barriers for these treatments. The satisfaction surveys were based on the Treatment Satisfaction Questionnaire for Medication (TSQM), which has been validated using a variety of medical conditions (1), and the Satisfaction with New Treatment for cavities, which was adapted from a survey for acceptance of SDF treatment in children (26). Findings indicate high levels of satisfaction with the SDF and ART treatments from the survey, and focus groups provided an in-depth explanation of why participants reported satisfaction and their perceived barriers to treatment outside of the study. During the survey collection and focus groups, the older adults may not have known if their caries were arrested or not, but we speculate that satisfaction may have been influenced by their overall perception of getting dental care and experiencing improved pain and sensitivity. Treatments were delivered with ease, reduced dental anxiety, and were convenient. Dissemination and willingness to receive future treatments were perhaps prompted by treatment location and benefits such as no needles or drilling. Finally, barriers and limited resources were reported for future dental care outside of the study.

Treatment satisfaction has previously been shown in younger age groups for SDF (18) and ART (17). Both survey and focus group data provided a better understanding of why older adult participants were satisfied with these treatments. *First*, in satisfaction surveys, older adults reported that they were satisfied with the treatment's ability to treat and prevent their caries. This was the participants' perception and was further supported in a focus group with one participant stating that they had a reduction in pain. A previous study also hypothesized that therapeutic benefits of SDF may be associated with satisfaction, which is consistent with our results, and high levels of satisfaction were reported with SDF despite the side effect of staining (18). In focus groups, satisfaction was further validated as treatments were described as painless and reduced dental anxiety. In a previous study, 90% of adults 60 years and older had dental anxiety, and fear of pain during procedures was a common reason for not seeking care (8), which makes SDF or ART caries treatment appropriate for those with dental anxiety. *Secondly*, survey responses indicated that treatments were easy or very easy to receive by the majority of participants, and focus group participants stated that treatment was convenient since it was delivered at their housing site, which alleviated transportation barriers. These results may be an indication that mobile dentists or dental treatment in a community setting is needed to reach this population. *Third*, another treatment advantage reported in focus groups was that the treatment was free. Many older adults lack dental insurance (4), and SDF and ART are much more affordable than conventional restorations and have been previously described as low-cost (11, 15). In fact, SDF and ART treatments are 3 times less of the cost of conventional restorations (37). Similar to our participants, in a non-US study, 83% of older adults reported that income, dental health issues, and dental fear influenced their intention to choose SDF treatment (33).

The findings of this study gave insight to dissemination factors and willingness to receive SDF or ART as future caries treatments. *First,* participant surveys indicated that over 90% were more likely to choose SDF or ART over restorative treatments requiring needles or drilling, and focus group responses reinforced these findings. This proportion is notably higher than a previous study, where 54% of parents reported that they were likely to choose SDF treatment for their children's posterior teeth over conventional restorations requiring drilling or needles (26). In the same study, acceptance rates increased with treatment and behavioral barriers (26); however, staining remained an influential factor for children (26), which was not observed in the present study with older adults. Second, all focus group participants stated that they would recommend these treatments to other older adults, because they were pain free and convenient. Similarly, in a previous study evaluating treatment satisfaction for rheumatoid arthritis using the TSQM, higher levels of satisfaction were reported in the convenience domain with a medication administered orally vs. by injection (2). This reinforces the importance of convenience and minimizing pain or discomfort with treatment, as reported with SDF and ART in this present study. Finally, in a prior study, discoloration of the anterior teeth in older adults influenced SDF treatment decisions (14). On the survey, side effects did not affect satisfaction with SDF treatment for the majority of participants, and dissatisfaction with SDF due discoloration was not reported in focus groups. In fact, only 7 participants declined SDF treatment at baseline for anterior teeth due to discoloration, with only three refusing at the follow-up visit. This is consistent with a previous study with adult patients, as SDF staining was considered acceptable to 85% of the participants due to the benefits of the treatment (18).

Barriers and resources were only addressed in focus groups. The majority of the older adults reported some type of barrier and limited resources for dental care outside of the study, which was consistent with previously reported barriers including affordability, transportation, and poor dental experiences (6, 7). Notably, a large portion of focus group participants said that they had not visited the dentist in over a year (e.g., years), with one stating a lack of motivation as the reason. Motivation can be a barrier in maintaining good oral hygiene in older adults, which may increase the risk of developing dental caries (38). For this study, no barriers for SDF or ART treatments were reported, with one exception of scheduling during COVID. Resources were limited among participants for future dental care, although few reported facility (i.e., social workers, transportation), family, and dental insurance or payment plans as resources.

These barriers and limited resources emphasize the need for dissemination of these findings to improve access to SDF and ART in dental, medical, and community settings. Continuing Education programs could be offered for dentists that report the results of this study and the feasibility of SDF and ART treatments, especially for patients with conditions such as dental anxiety, disabilities, or mobility challenges. Additionally, medical providers may apply SDF at office visits since a billing code for this treatment was approved by the American Medical Association (12). This would allow for caries treatment during a primary care visit for those that were not able to visit a dentist due to insurance, dental anxiety, or transportation barriers. Also, a Continuing Medical Education program could be used to teach medical providers these techniques and billing information for medical staff. For community settings, a dental or medical provider may offer this type of treatment during periodic mobile health visits as a way to reach older adults in the community.

There are limitations to this study. One limitation is that participants completed satisfaction surveys with the research team which may have introduced social desirability bias. Focus group findings may also be subject to recall bias as some participants may have completed treatment several months prior to the discussion. This study did use a moderator who had no prior interaction with participants during focus group discussions to minimize bias and is a strength of this qualitative data collection.

## 5 Conclusion

This study explored satisfaction and experiences with SDF and ART caries treatments among older adults, which were successfully delivered in a community setting. Older adults preferred these minimally invasive treatments to conventional restorations that use drilling and local anesthesia.

## Data Availability

The data supporting the conclusions of this article will be made available from the corresponding author upon reasonable request.

## Author's note

The statements in this article are solely the responsibility of the authors and do not necessarily represent the views of the Patient-Centered Outcomes Research Institute (PCORI), its Board of Governors or Methodology Committee.
