# Supplementary material for: Community-dwelling older adults’ satisfaction and experiences with two non-surgical caries treatments: a mixed methods study
Source: Front Oral Health. 2026 May 11;7:1814583. doi: 10.3389/froh.2026.1814583 (PMC13199272; doi:10.3389/froh.2026.1814583)
Supplement: Supplementary file 1 [file Table1.docx]

Supplementary Material

# Supplementary Data:

**PCORI Focus Group Interview Guide:**

Good Morning and Welcome to this Focus Group Discussion sponsored by CWRU.

We thank you all for taking the time today to participate in our discussion.

My name is…and I will be the moderator for today’s discussion.

Our discussion today will be about your experience with the DENTAL TREATMENT that you received in our study. Some of you received the Atraumatic Restoration Treatment (ART) with Fluoride Varnish (FV) to all teeth; and some of you received the Silver Diamine Fluoride (SDF); and all of you have completed the study treatments.

I would like to encourage all of you to participate freely in our discussion – there are no right or wrong answers, and we would like to hear as many of your stories and experiences as possible within the hour of our discussion.

Also, if you would like to answer a question discussed earlier but we have already moved on to another topic, please do not hesitate to share your thoughts. Your comments will help us to know your experience and satisfaction with our dental treatment interventions that were used to treat dental cavities; and the information you share will help us to plan for its use in community settings.

Let me begin by sharing some GROUND RULES so that we all understand what is going on. This is a research project, and we will not be trying to sell you anything. We would prefer that one person talk at a time; and if you would talk loud enough so that we can all hear you, that would be great!

We are audio recording this discussion so that we do not miss anyone’s comments. We will address each other by our first names during the discussion, and your names will be removed from the report later to maintain confidentiality. All of your comments are completely confidential and will be used for research purposes only. We would like to hear both positive as well negative (if any) experiences.

Do you have any questions about the arrangement, before we start?

If NO, we shall start with the discussion now.

QUESTION 1:

Please describe your EXPERIENCE and SATISFACTION levels with dental treatment (SDF or ART +FV) that you received in this research study?

PROBES:

Did you feel OK after the dental treatment?

Were you satisfied with the dental treatment that you received (SDF/ART + FV)

If you have a cavity in the future, would you be willing to get the same dental treatment that you received in our study?

QUESTION 2:

Having received dental treatment with SDF and ART + FV from our research study; would you RECOMMEND these treatment options to other older adults such as yourself?

PROBES:

What are some of the positive and/or negative aspects of getting this type of dental treatment (for you or any other older adult)?

In general, what do you think older adults in your community need in terms of oral health and dental care?

QUESTION 3:

What were the BARRIERS and CHALLENGES that you faced in getting our research project related dental treatment?

PROBES:

Did you feel any pain or discomfort while getting dental treatment?

Did you have to wait for a long time to schedule an appointment with us at your facility?

Did it take a long time in the dental chair while getting our study treatment?

TRANSITION NOTE: So far we have discussed about your experiences with our dental research project. We thank you for successfully completing the study participation. Some of you may have received referrals from our study for future dental care; so going forward ……

QUESTION 4:

What would the BARRIERS and CHALLENGES be for you or other older adults in your community to receive dental treatment?

I am going to share my screen and write down the Barriers & Challenges that you mention; so that we can see what you have said.

PROBES:

Issues related to Convenience (e.g., accessibility, transportation, etc.); Communication (not knowing where to go or what treatment options are available, etc.)?

QUESTION 5:

What are the RESOURCES available to you to seek dental care in the future?

Again, I will write down the Resources that you mention; so that we can see what you have said.

PROBES:

Do you know about dentists who accept adults enrolled in Medicaid?

Do you know that Ohio Medicaid provides coverage for adult’s dental care?

Do you know what dental services are covered?

Does your housing facility provide resources that address any of the challenges we listed?

Does your housing facility provide any of the following resources: Signing up for Medicaid, navigating the system, finding transportation options, or scheduling dental appointments for you?

SUMMARY & WRAP-UP:

Is there anything else, anyone would like to add to the discussion before we wrap-up?

Is there anything else that the research team members would like to add to this discussion?

In Summary, we have discussed about your experiences and satisfaction with the dental treatments that you received in our study and if you would be willing to recommend this type of dental treatment to other older adults; we spoke about the barriers and challenges that you faced in getting this dental treatment from our research study. We also discussed about the potential barriers and challenges that you or other older adults in your community, may face to receive dental treatment or dental care in the future; and you have mentioned the resources that are available to you to seek dental care in the future.

On behalf of our study team, I would like to thank you for taking part in the focus group discussion.

Your views and inputs are valuable, helpful and contribute to our future intervention study.

If you have anything you would like to add or have any further questions, please do not hesitate to contact us.

After this call, please contact our Dental Study staff at your facility; to receive your boxed lunch and payment.

Thank you everyone!

Have a wonderful day!

Goodbye.
